# Trevor Mann (1916–1996): Paediatrician responsible for the development of hospital services for children in Brighton, England

**DOI:** 10.1177/09677720221090876

**Published:** 2022-04-04

**Authors:** Rosemarie Patterson, Sangeetha Sornalingam, Maxwell John Cooper

**Affiliations:** 152127 Department of Primary Care and Public Health, 12190 Brighton and Sussex Medical School, Brighton, UK

**Keywords:** Paediatrics, medical history, biography, Trevor Mann, Brighton

## Abstract

Trevor Philip Mann (1916–1996) was the first consultant paediatrician at the Royal Alexandra Children's Hospital (RACH) in Brighton, since its foundation in 1881. Here, he was responsible for significant service developments, including establishing a department of paediatric surgery and the first neonatal unit in England outside of London. Mann grew up in South London, and aged 14 had a lengthy admission to hospital with tuberculosis. He studied medicine at St Mary's Hospital, London. During World War II he was a Royal Navy Surgeon-Lieutenant, aboard the Atlantic destroyer, HMS Georgetown, and with the Russian convoys, before completing paediatric training in London. Here, he was involved in treating paediatric tuberculous meningitis; clinical work that formed part of one of the earliest randomised controlled trials. In 1951 Mann moved to the RACH where he researched infantile infectious gastroenteritis and introduced (now commonplace) practices at the hospital, including barrier nursing. He lived in Rottingdean, Sussex, and enjoyed sailing, gardening and wood turning. Mann's impact on paediatric care in Brighton was recognised by the hospital, naming the Trevor Mann Baby Unit in his honour, upon his retirement in 1981. This article seeks to record his contributions and reconnect local clinicians with his memory.

Trevor Philip Mann (1916–1996) was the first consultant paediatrician at the Royal Alexandra Children's Hospital (RACH) in Brighton, England. He was responsible for founding the hospital's department of paediatric surgery and the first neonatal unit in England outside of London. In 1981, this unit was renamed the Trevor Mann Baby Unit, in recognition of his lifetime of dedication towards paediatric care, service development and academic writing.

## Early life and war years

Born on 29th September 1916 in Wandsworth, Greater London, Trevor was the oldest child of Gertrude and Francis Mann.^1^ His mother, Gertrude Violet Cogan, was born in 1887 in Hampshire^1^; prior to marrying Francis in 1915, she was a nursery governess, and gave up regular employment following the birth of Trevor.^1,2^ His father, Francis Mann, was born in Fulham in 1881 and worked as an insurance broker.^1,3^ Trevor's sister Pat was born in 1921,^1^ and went on to become a nurse.^4^ During the early part of his life, Trevor and his family lived in Mitcham, later moving to Norwood, both located in South London.^5,6^ At age fourteen, Trevor became unwell with tuberculosis (TB), and spent almost a year in hospital. This may have shaped his later decisions to both study medicine and to contribute to clinical research into the treatment of TB.^4^

Trevor commenced his medical studies at St Mary's Hospital in London in 1936, qualifying in 1941.^7,8^ At the onset of war in 1939, he was on a student placement in Park Prewett Hospital in Basingstoke.^3^ Following a short period working at Hammersmith Hospital, Trevor joined the Royal Naval Volunteer Reserve in 1942, as a Temporary Surgeon Lieutenant.^9,10^ He remained on the navy list until 1946.^9^ During this time, he served on the Atlantic destroyer HMS Georgetown and, with the Russian convoys, including aboard the aircraft carrier HMS Campania.^4,11^ Trevor married Joyce Iris Ladbrook in 1946, who he met while she was serving in the Women's Royal Naval Service (the Wrens) in Northern Ireland.^1^ They lived together in South London, settling in Peckham in 1949.^12^

## Paediatric training

On returning to London after the war, Trevor returned to work in Hammersmith as a paediatric house doctor.^10^ Shortly after, he took on the role of Streptomycin Registrar for the Medical Research Council from 1946–1948.^4,13^ In this capacity he was involved in the early use of streptomycin to treat TB meningitis.^13,14^ TB meningitis had previously been fatal, however Trevor oversaw treatment via intra-muscular and intra-thecal routes, leading to some cases of cure.^13,15^ The initial study is considered one of the first intentional randomised controlled trials, with the epidemiologist Sir Austin Bradford-Hill (1897–1991) involved in its design.^14,16^

Following this, Trevor moved to London's Great Ormond Street Hospital, where as a registrar he became first assistant to Sir Alan Moncrieff (1901–1971), who at the time was the first Professor of Child Health at the University of London.^17–19^ Moncrieff was responsible for developing England's first premature baby unit which opened in Hammersmith Hospital in 1947.^18^ Trevor spent a significant portion of his time working in this department, perhaps shaping his later interest in neonatology.^18^

## Early years in Brighton

In 1951, Trevor was appointed as the first consultant paediatrician at the Royal Alexandra Hospital for Sick Children in Brighton, known locally as ‘the Alex’.^11^ The Alex had opened in 1868, however prior to Trevor's employment, had been staffed by resident medical officers and honorary medical staff (most of whom were adult physicians).^19,20^ The Alex had recently been expanded, and in 1951 housed several medical wards, including one with separate rooms for infectious patients.^20^ Trevor was initially contracted for six sessions (the equivalent of three days) per week, and also spent a significant portion of time working in peripheral clinics around Sussex.^19^ As the only consultant in the hospital, Trevor was regularly on-call and frequently attended the hospital for emergency work. This included carrying out exchange transfusions for children with Rhesus disease, which was common at the time.^4^

Trevor also continued to work closely with the London hospitals. Initially he travelled up to London himself, however after just a few years he began persuading specialists from London to travel down to Brighton to deliver clinics with him in the area.^10^ Trevor also managed many children with polio during the early years in Brighton (in the mid-1950s); as his own children were therefore at high-risk of catching the virus, they were amongst the first to receive polio vaccination in Sussex.^4,19^

Perhaps resulting from his own childhood stay in hospital (when parents were only able to visit for 1 hour each week), Trevor introduced parental open access policies at the Alex, encouraging mothers to be in hospital with their unwell children.^4,20^ He worked with Dr Dermod MacCarthy, a paediatrician in Aylesbury, Buckinghamshire, and someone Trevor had worked with in Hammersmith, to encourage other hospitals to also introduce this change nationwide.^4,21^

Trevor continued his interest in research, despite significant clinical commitments. In the early years of his time at the Alex, Trevor particularly focused on the field of infantile infectious gastroenteritis, publishing a paper on this in The Lancet in 1954.^22^ In this work, Trevor recognised the importance of preventing the spread of hospital acquired infection and introduced a strict hand washing regime to prevent transmission.^22^ He was also responsible for introducing additional measures such as barrier nursing and pre-heating feeds to prevent spread.^22^ He redesigned a ward at the Alex to include cubicles, to help prevent spread of infection further.^23^ These changes were associated with a decline in cases of infectious diarrhoea at the hospital.^22^ His innovations helped to raise the profile of the Alex, largely contributing to its reputation as an excellent hospital to train and work in.^20^

During his early time working at the Alex, there was no provision for neonatal care at the hospital. Trevor therefore took sabbatical time during the 1960s, spending this at University College London Hospital learning practical and theoretical skills around caring for neonates and the use of ventilators.^4^ Following this, he established a neonatal unit at the Alex in the 1970s.^11,24^ This was the first of its kind in England, outside of London.^11,24^ The neonatal unit would later be renamed the Trevor Mann Baby Unit on his retirement in 1981, in recognition of his contribution to neonatal care in Brighton.^11,19^ Trevor also published widely in the field of neonatology, covering topics such as neonatal hypothermia, hypoglycaemia and sudden death in infancy.^25–27^

## Wider work

Amongst his other research interests, Trevor published several case reports, detailing rare diagnoses and unusual presentations.^28–30^ These included siblings presenting with symptomatic hypernatraemia following excessive salt intake at home, and a case of familial pancreatic exocrine dysfunction which had previously been undiagnosed.^28,30^ He was also one of the first to recognise the importance of fundoscopy to aid assessment in cases of suspected physical child abuse, a practice which is now routine.^31^ Later in his career, Trevor also published the Colour Atlas of Paediatric Facial Diagnosis in 1989, using clinical images he had collected over the course of his career and from colleagues.^32,33^ Its review offers the following description:

“This book, compiled by a keen clinical observer, will enrich the repertoire of the practised paediatrician and should serve as a stimulant and diagnostic aid to our more junior colleagues. Here we have richly illustrated material presented by one experienced and prodigiously energetic man [i.e. Trevor Mann] who has sought to fit his clinical cameos with the perfect photograph …*”*.^34^

The review goes on to praise Trevor for recognising the importance of observation as an essential clinical skill and for the breadth of diagnoses covered, particularly within neonatology.^34^ Trevor was clearly well liked by the author, David Baum, who was Professor of Child Health at the Royal Hospital for Sick Children, Bristol. Trevor's sense of humour is hinted at in the review, explaining that images from “*friends and colleagues, who have shared his passion for clinical observation and photographic record*” are displayed with “*the name of the photographer prominently, suggesting to future medical historians that these were the facial characteristics of Peter Dunn, Cyril Chantler, Martin Moncrieff, Neil O’Doherty, Maclom Chiswick, Roy Meadow* et al.*!*”.^34^ This “*whimsical turn to the atlas*” highlights the positive, collaborative relationships Trevor had with his contemporaries.

This is also recognised by Dr Geoffrey Hatcher, who worked with Trevor as a consultant paediatrician at the Alex from 1970–1992. He remembered Trevor in his eulogy (which he read at Trevor's funeral), with the following words:

“Neither was he an easy master. He expected and demanded a great deal from his nursing staff, the secretaries who worked with him, his house physicians and his registrars, not to speak of his consultant colleagues. But he repaid them all in full measure by his teaching, his support and his kindness.”^19^

These qualities aided Trevor in attracting a range of aspiring trainee paediatricians to the Alex.^19^ He was well recognised as an excellent teacher and helped support colleagues in their own research endeavours.^19^

Trevor also oversaw the development of a paediatric surgery division at the Alex, where previously children with surgical conditions had required transfer to surgical centres in London or Southampton.^4,11,24^ He also worked as a locum doctor in Gibraltar regularly in the 1970s, covering the holiday leave of the only paediatric doctor serving the population.^4^ During one of these stints, he organised the transfer of an unwell premature baby to London, courtesy of the Royal Air Force.^4^ The flight was extended as it had to take place just above sea level to maintain optimal oxygen levels, however the baby was safely transferred to Queen Charlotte's Hospital in London and survived.^4^

Trevor was also responsible for founding The Royal Alexandra Centenary Appeal for Research in 1968.^20^ This was initially set up with the aim of raising money to fund a ventilator for the Alex but also helped to fund much of the emerging research at the hospital. The charity is now known as Rockinghorse Children's Charity, and is the official charity of the Alex.^35^

In addition to these achievements, Trevor regularly attended meetings of the British Paediatric Association; he later served as a member of the council.^36^ Trevor also regularly presented at the Royal Society of Medicine meetings. In 1974 he became president of its Section of Paediatrics; his inaugural address discussed a history of toys.^37–39^ His interest in toys extended to developing a toy library in Brighton for children with neurological disabilities.^4,11^ Trevor also acted as an adviser to the Department of Health and Nutrition and sat on expert committees created by the Royal College of Physicians, London, where he was also an examiner.^19^ From 1973, Trevor was given the role of Honorary Tutor in Paediatrics at Guy's Hospital and Medical School.^19^ He also became a Visiting Fellow at the local University of Sussex.^19^

## Family and personal life

Trevor was father to four children. His son Nicholas Mann became a paediatric surgeon himself, in Reading.^11^ Trevor continued to sail throughout his life, initially dinghy sailing locally in Shoreham, Sussex.^4^ Later, Trevor sailed in larger boats to France, Scotland or Cornwall, where he enjoyed shark fishing.^4^ Later in his life he spent time as a doctor on cruise ships during holidays and teaching others interested in learning to sail.^10^ Trevor enjoyed travel more widely and drove his young family on holidays to France, Portugal and Spain.^4,19^

Throughout his life, Trevor was a keen gardener, even planting a small vineyard in Rottingdean, Brighton, in the 1970s and 1980s, that produced around 40 bottles of wine each year.^4,10^ He was also interested in woodwork; on his retirement he was gifted a lathe, leading to him becoming a talented wood turner.^10,11^ He furthers contributed to his local community furthers, through being an active member of the Rottingdean Preservation Society.^19^ Trevor died of prostate carcinoma on 24th September 1996, days before his 80th birthday.^1,11^

## Conclusion

Trevor Mann was the first consultant paediatrician in Brighton and responsible for advancing paediatric care in the city and region. He was specifically responsible for founding and developing paediatric neonatal and surgical services in the area. His decision to become a paediatrician and his focus on patient care appears to have been shaped by his personal experiences of illness as a child. His decision to work in Brighton may have been influenced by his apparent affinity with the sea. He is fondly remembered by retired colleagues, with recollections of his work ethic and high clinical standards shining through. In recognition of his achievements, his name has been memorialised in the Trevor Mann Baby Unit. Twenty-five years after his death, it is opportune to remember his life and it is hoped that this work will serve to promote his legacy amongst younger colleagues in Sussex and, in particular, at the Alex today.

Included with submission are four photographs, attached separately:

- Two of Trevor Mann – provided by Nicholas Mann

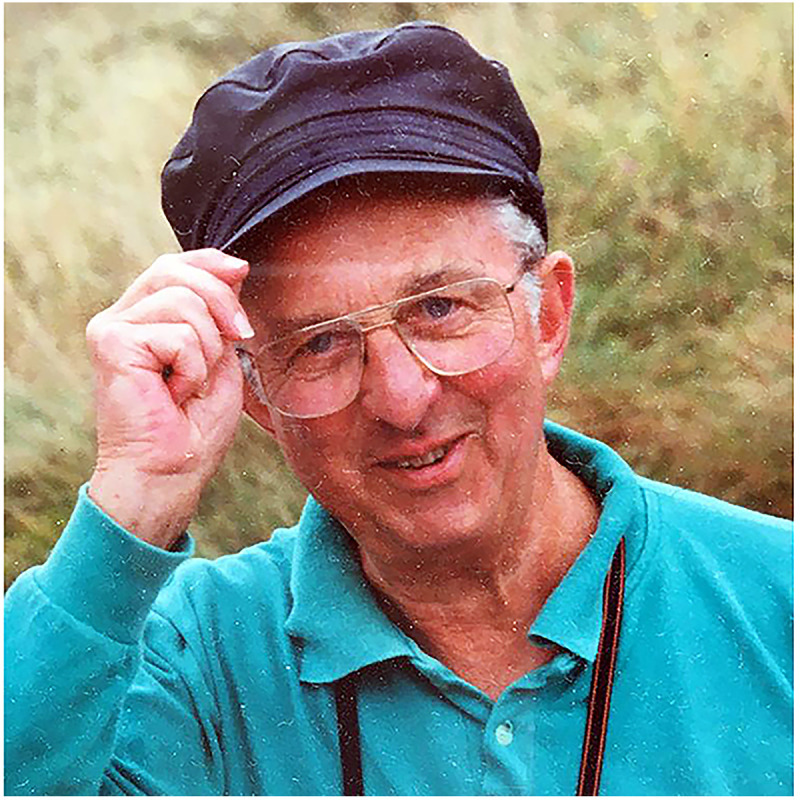

  Dr Trevor Mann. Courtesy of Dr Nicholas Mann.

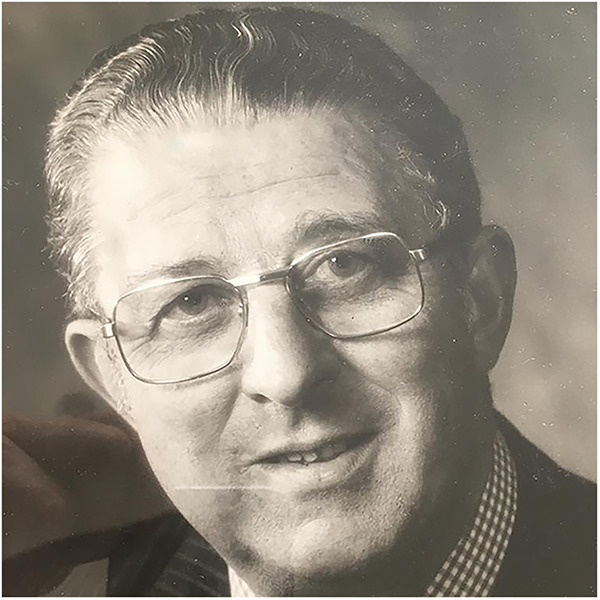

  Dr Trevor Mann. Courtesy of Dr Nicholas Mann.
- One photo of Trevor's house in Rottingdean – taken by Rosemarie Patterson, August 2021
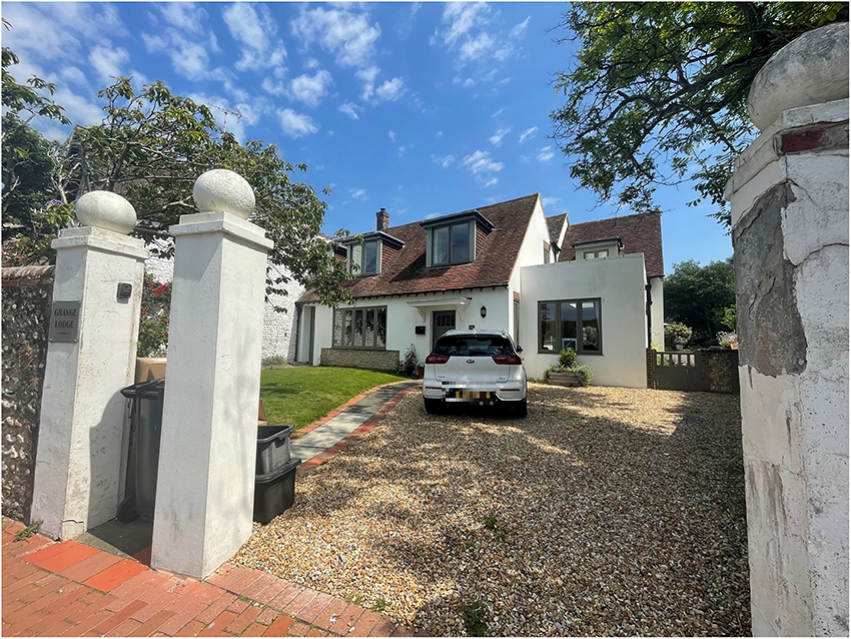
The house in Rottingdean. Photograph taken by Rosemarie Patterson, August 2021.
- One photo of the RACH – taken by Rosemarie Patterson, November 2021
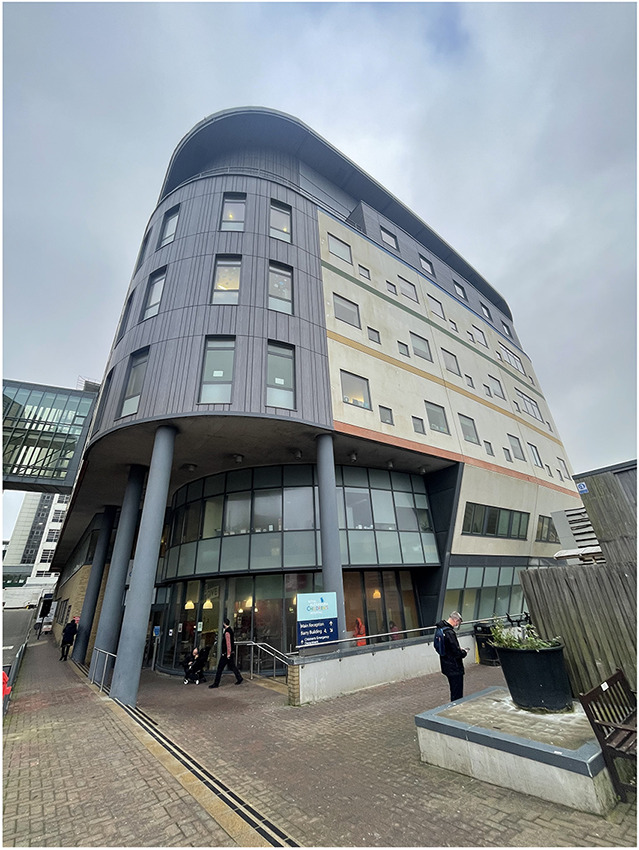
The RACH. Photograph taken by Rosemarie Patterson, November 2021.
